# Preparing Laboratory and Real-World EEG Data for Large-Scale Analysis: A Containerized Approach

**DOI:** 10.3389/fninf.2016.00007

**Published:** 2016-03-08

**Authors:** Nima Bigdely-Shamlo, Scott Makeig, Kay A. Robbins

**Affiliations:** ^1^Qusp Labs, Qusp, San DiegoCA, USA; ^2^Swartz Center for Computational Neuroscience, University of California, San Diego, San DiegoCA, USA; ^3^Department of Computer Science, University of Texas at San Antonio, San AntonioTX, USA

**Keywords:** EEG, BCI, large scale analysis, neuroinformatics

## Abstract

Large-scale analysis of EEG and other physiological measures promises new insights into brain processes and more accurate and robust brain–computer interface models. However, the absence of standardized vocabularies for annotating events in a machine understandable manner, the welter of collection-specific data organizations, the difficulty in moving data across processing platforms, and the unavailability of agreed-upon standards for preprocessing have prevented large-scale analyses of EEG. Here we describe a “containerized” approach and freely available tools we have developed to facilitate the process of annotating, packaging, and preprocessing EEG data collections to enable data sharing, archiving, large-scale machine learning/data mining and (meta-)analysis. The EEG Study Schema (ESS) comprises three data “Levels,” each with its own XML-document schema and file/folder convention, plus a standardized (PREP) pipeline to move raw (*Data Level 1*) data to a basic preprocessed state *(Data Level 2*) suitable for application of a large class of EEG analysis methods. Researchers can ship a study as a single unit and operate on its data using a standardized interface. ESS does not require a central database and provides all the metadata data necessary to execute a wide variety of EEG processing pipelines. The primary focus of ESS is automated in-depth analysis and meta-analysis EEG studies. However, ESS can also encapsulate meta-information for the other modalities such as eye tracking, that are increasingly used in both laboratory and real-world neuroimaging. ESS schema and tools are freely available at www.eegstudy.org and a central catalog of over 850 GB of existing data in ESS format is available at studycatalog.org. These tools and resources are part of a larger effort to enable data sharing at sufficient scale for researchers to engage in truly large-scale EEG analysis and data mining (BigEEG.org).

## Introduction

Traditional laboratory EEG studies typically collect data from a relatively small number of subjects in a controlled environment, using paradigms including a limited number of instances of clearly defined experimental event types, subject tasks, and task conditions. For many such studies, the original researchers may have only extracted a few simple measures such as event related potential (ERP) peak amplitude(s) at one or more scalp channels or mean EEG spectral power over some scalp region. Carefully collected datasets from 10s of 1000s of such experiment sessions currently reside on disks in individual investigators’ laboratories. Unfortunately, they become unusable once the many details needed to process them meaningfully have been lost or forgotten. This data, collected at great expense and effort, could be analyzed collectively using rapidly evolving statistical data modeling methods to shed new light on the cortical brain dynamics that support a range of cognitive abilities in both healthy and unhealthy subjects. The common availability of such data or of measures derived from it could allow other researchers to evaluate their results within a broader context of existing data and data measures.

In the medical arena, an increasing recognition of the significant inter- and intra-subject variability in EEG, plus the variety of possible environmental interactions that might meaningfully affect brain dynamics, point to large-scale data analysis as a way to examine the generalizability of analysis results across subjects and paradigms. For example, the Research Domain Criteria (RDoC) initiative of the US National Institute of Mental Health (NIMH) seeks to find common deficits across subjects given different psychiatric diagnoses by focusing more on the nature of individual differences in both cognition and brain dynamics across both normal and abnormal subject populations (Cuthbert and Insel, 2013).

Real-world brain imaging using EEG supported by physiological monitoring technologies in less controlled conditions (Liao et al., 2012; Ortiz-Rosario and Adeli, 2013) aims to create brain–computer interface (BCI) classifiers that operate robustly in daily life while offering opportunities for learning how human brain processes operate in more ecologically valid environments and conditions. One way BCI research can counteract the heightened variability introduced by real-world conditions is by performing larger-scale modeling and analysis, if larger-scale collections are available. Transfer learning methods (Fazli et al., 2009; Alamgir et al., 2010; Lotte and Guan, 2011) transfer knowledge gained from data acquired under one set of conditions to improve the classification of data recorded under other conditions. Improved BCI classification accuracy and reduced calibration data requirements have already been demonstrated using such approaches (Lotte, 2015). However, while these methods can potentially produce the greatest performance improvements from application to large-scale data collections, to achieve a level of prediction accuracy needed for real-world applications these must consist of well-specified, “interoperable” data sets of sufficient size and variety.

As the impetus grows to pool EEG data acquired under a variety of experimental conditions and studies, several difficulties emerge from lack of standards for characterizing data events and experimental conditions, and organizing the data files constituting each collected study. A scientist wishing to incorporate data from another laboratory (or their own previous grant cycle) into a new analysis or meta-analysis must often manually identify events of interest from the specific and often cryptic event codes originally assigned by the experimenters, often by sifting through the experimenter’s notes and published papers for relevant information about the data. Beyond the problem of translating event annotations and experimental conditions into a common format, the researcher must worry about organizing the recording files and their associations with recording sessions, subjects and subject-related information (e.g., subject demographics, medication, handedness, group, etc.). These associations become more complicated in experiments including EEG plus additional data streams such as body motion capture and eye tracking. Lastly, recordings made in real world conditions and/or in longer recording sessions are prone to many more “technical errors” and typically include more non-brain EEG sources (i.e., artifacts) than data from shorter and/or more tightly controlled laboratory sessions. Furthermore, during the initial data analysis process the original experimenters are likely to have produced multiple versions of the data, each preprocessed in some loosely documented manner. Thus, data analysis or meta-analysis beyond the scale of a single EEG study is still rare.

Although the tools for analyzing and sharing neurophysiological data are evolving, there is a gap between current capabilities of data sharing tools and the practical requirements for larger-scale automated analysis of EEG data. In a typical EEG experiment, the raw data undergo a number of processing steps that are usually only documented in the experiment’s notes, computer scripts, or memory. Even when careful researchers fully document these items, each data set will require considerable manual curation, cleaning, and reformatting to be of use in new, larger-scale analysis efforts.

We have embarked on the process of building an open infrastructure and tools [summarized in Appendix A (Table A1)] in a collaborative effort we term BigEEG. As a part of our effort to tackle the lack of EEG data standardization and consistency, we have developed a *containerized data* approach with several levels of standardization and a supporting infrastructure to make EEG study data portable, searchable and extractable. This containerization does not preclude the use of other metadata standards, but rather emphasizes curation, data quality, and uniformity so that researchers can apply automated machine learning techniques to their own and/or others’ data with no or only a minimum amount of manual curation.

Many fields have successfully employed data containerization to provide simplified and portable interfaces to unstructured or loosely structured data ‘payloads’ in the same way that intermodal shipping containers have revolutionized global commerce by replacing “break bulk” and other modes of transportation that required lengthy, manual handling of cargo (Levinson, 2008), ‘Virtual Machine’ containers have enabled and are now an important basis for cloud computing. Most recently, Linux^1^, and in particular Docker software application containers^2^, have gained significant attention in the software development world. These technologies wrap an application in a complete file system containing code, tools and all the dependencies, e.g., runtime libraries, needed to run the application (Pahl, 2015).

The data containerization approach proposed in this paper refers to organizing data and metadata for an EEG study using a standardized file structure and metadata encapsulation schema. Unlike application containers provided by Docker, ESS only provides data containerization (no executable applications). Researchers can ship a data collection as a single unit and operate on its data using a standardized interface without having to deal with idiosyncrasies of its organization. This paper describes the ESS data standardization pipeline and explains the reasoning behind it. We detail tools we have made freely available, under the MIT license, for researchers to use on their own data or to more easily incorporate containerized data from other researchers into their research. We also briefly report on our experience of using these tools to work on a number of EEG data studies recorded using various EEG systems in a number of laboratories.

## Materials And Tools

### State of the Art

The tasks required to prepare EEG studies for data sharing and large-scale analysis include: (1) describing the experimental paradigm and experimental events of interest in a standardized manner; (2) encapsulating study-level metadata (subject groups, association of files, subject and sessions, data provenance, etc.) in a standardized manner; (3) providing access to online resources allowing users to find and download the data. Several efforts relevant to EEG data sharing address one or more of these tasks.

One of the oldest and most well-supported data repository efforts is PhysioNet (Goldberger et al., 2000), which consists of freely available tools and downloadable data archives. Although PhysioNet does have some EEG collections, its primary focus is electrocardiographic (ECG) recordings. The metadata and event documentation provided in PhysioNet consists only of experimenter text notes which are not standardized across collections.

G-NODE (Sobolev et al., 2014) provides a full data management system along with supporting tools for access, data-sharing, and analysis. The G-NODE (Sobolev et al., 2014) odML data model (Grewe et al., 2011) provides support for organizing study metadata as key-value pairs. The companion Neo data format (Garcia et al., 2014) provides a flexible method of manipulating arbitrary physiological data. The primary focus of G-NODE, however, is cellular and systems neurophysiology; G-NODE does not address the issue of standardized event annotation. The Brain Imaging Data Structure (BIDS) (Gorgolewski et al., 2015) is a recent effort to create a new standard for describing and organizing human neuroimaging data. BIDS has many features in common with our ESS including specification of a standardized file/folder structure and file-based metadata encapsulation^3^. However, BIDS is focused on fMRI data. Other efforts for sharing fMRI data include OpenfMRI (Poldrack et al., 2013) and NeuroVault (Gorgolewski et al., 2015).

More general efforts to support neuroscience data sharing include INCF^4^, XNAT (Marcus et al., 2007), and COINS (Scott et al., 2011). The INCF Dataspace provides a clearinghouse for sharing neuroscience resources. XNAT is an informatics platform for managing, exploring, and sharing neuroimaging data that emphasizes MRI and fMRI data. COINS, a similar system, emphases fMRI and MEG data and uses a more distributed model for dealing with protected health information (PHI). Neuroimaging Informatics Tools and Resources Clearinghouse (NITRC) is an NIH-funded online resource that facilitates finding and comparing neuroimaging resources (tools and data) for functional and structural neuroimaging analyses (Luo et al., 2009).

In the bioinformatics field, the ISA-Tab format (Sansone et al., 2012) enables rich descriptions of experimental metadata to facilitate reusable data and reproducible results. ISA-Tab comes with an extensive set of tools for creation, validation and conversion to linked-data^5^ and is built upon MAGE-TAB (Rayner et al., 2006), a tab-delimited format for exchanging microarray data. Several domain-specific bioinformatics formats (such as ‘ISA-Tab nano’ for nanomaterials, and SNRNASM for Single Nucleotide Resolution Nucleic Acid Structure Mapping) are built on ISA-TAB.

There exist a number of resources for general hosting and publication of scientific data. SeedMe (Chourasia et al., 2013) is an NSF-funded project promoting general data sharing with metadata and cloud-based visualization capabilities. A similar resource for storing and publishing scientific data is FigShare^6^ (Singh, 2011), which provides features such as digital object identifiers (DOIs) (Paskin, 2008) for published data and fine-grained “embargo” for automatically making private data public after a predefined time period. Dryad (White et al., 2008) is another solution for long-term scientific data publication, also offering DOI assignment and embargo capabilities.

While EEG data are at best a secondary consideration in the above efforts, EEG data sharing and comparison is the primary focus of projects including NEMO, the EEGBase EEG/ERP portal, and HeadIT. The NEMO project (Dou et al., 2007) provides an ontology, a set of tools and a centralized database for raw EEG and ERPs with an overall goal of better categorizing and labeling ERP features. The EEG/ERP portal (EEGBase) (Mouček et al., 2014) is a web-based system^7^ that allows users to upload, download, and manage EEG/ERP data. The system provides some workflow and group management capabilities and focuses on the semantic enrichment of ERP data (Jezek et al., 2011). It thus provides extensive tools for mapping between semantic representations (Ježek and Mouček, 2015). HeadIT^8^ makes available raw data from a number of event-related EEG studies.

Some observations in regards to the current state of the neuroinformatics resources:

(1) There are a relatively large number of resources for general-purpose hosting and publishing of scientific data (FigShare, SeedMe, Dryad).
(2) fMRI is the best-supported modality in terms of neuroinformatics technologies (XNAT, COINS, BIDS, OpenfMRI, NeuroVault).
(3) Resources supporting EEG data sharing and archiving have so far either focused on integrated ERP-centric analysis workflows with a limited experimental event ontology (NEMO), or (EEGbase) use generic data models (odML) without a comprehensive data schema tailored for larger-scale EEG analysis.

In formulating the requirements for a system that supports successful large-scale processing of EEG, we concluded that such a system must standardize study metadata and organization as well as event annotation in a machine-readable format. Furthermore, since most researchers read, process, and share data from single files in local file folders, such a system should be well suited to local, file-based processing pipelines. The system should also accommodate multimodal data and facilitate the shipment of data elsewhere for sharing or for taking advantage of distributed computing resources. There are already many for sharing scientific data in a generic, file-based manner (e.g., FigShare, SeedMe, Dryad) and a file-based metadata format provides researchers with the flexibility to choose any of these resources to share their data.

The efforts described in this paper, collectively termed BigEEG^9^, aim specifically to address this set of use cases with a particular focus on enabling automated processing of EEG studies. The BigEEG project focuses on ontologies/schemas and tools for documenting the nature of experimental events. The effort includes the extensible Hierarchical Event Descriptor (HED) system for describing the nature of experimental events whose standard and code are available at *www.hedtags.org* (Bigdely-Shamlo et al., 2013b), and the EEG Study Schema (ESS), which encapsulates EEG study metadata and is described below. Initial versions of these technologies were developed under the HeadIT project (Swartz Center for Computational Neuroscience, UCSD), which also hosts an online file-sharing resource^10^. **Table 1** compares BigEEG with the other technologies reviewed in this section in terms of specified requirements.

**Table 1:** Overview of EEG data technologies.

Effort	Focus	A/O	Mul	Meta	Event
BigEEG (ESS & HED)	EEG	Yes	Yes	ESS	HED
	EEG	No	No	odML OWL, RDF	No
G-NODE	Cellular and systems neurophysiology	odML	Yes	odML	No
PhysioNet	ECG	No	Yes	No	No
EEG/ERP portal	Raw EEG, ERP	Yes	Yes	odML	No
NEMO	Raw EEG, ERP	No	No	No	NEMO
INCF Dataspace	General	No	Yes	No	No
NITRC	General Neuroscience	No	Yes	No	No
CARMEN	Electrophysiology (cells)	No	Yes	MINI	No
BIDS	fMRI	Yes	Yes	NIDM-Experiment	No
XNAT	fMRI, MRI	No	Yes	Yes	No
COINS	fMRI, MRI	No	Yes	COINS DB	No
SeedMe	General	No	Yes	No	No
FigShare	General	No	YES	No	No
Dryad	General	No	YES	No	No

*In the column titles: A/O, available oﬄine (file system representation); Mul, multimodal data support (e.g., eye tracking, motion capture); Meta, study metadata ontology; Event, event description ontology used (No, none).*

### Containerization for Large-Scale Analysis

The BigEEG effort is organized around the idea of an *EEG study*, defined as a self-contained group of (possibly multimodal) EEG data sets recorded using one or more experiment paradigms. The EEG Study Schema (ESS) version 2.0^11^ specifies several levels of standardization and supporting infrastructure designed to make the data portable, searchable, and extractable.

**FIGURE 1:**
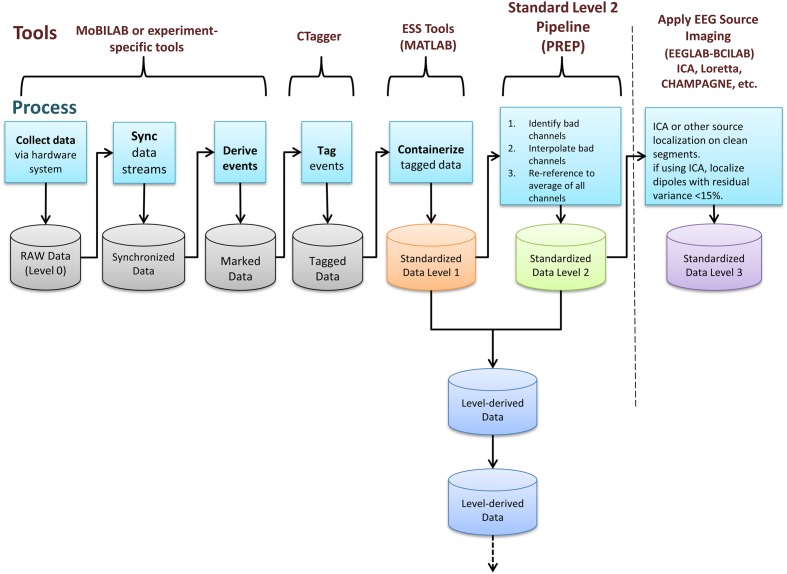
**EEG Study Schema (ESS) processing stages and tools used to transform data into successive containerized ESS standard level formats (Delorme and Makeig, 2004; Kothe and Makeig, 2013)**.

EEG Study Schema is built on the concept of data containers. Each container is a folder with a particular arrangement of files and a standardized XML descriptor file specifying study-level metadata. Containers are standalone entities that include all the information needed for a researcher to understand and process the data. A key result is that researchers can then develop fully documented, automated processing procedures using standardized container interfaces. This standardization means that code for processing HED/ESS data, once written, may be applied to any ESS-containerized study. ESS defines containers for data at different standard levels of processing. **Figure 1** shows these stages of data processing and tools used to transform data to ESS standards.

EEG analysis relies on identifying events and accurately marking their times of occurrence with respect to the EEG data. Researchers should derive experimentally relevant events from experiment control program logs and/or from the data themselves (e.g., occurrences of eye blinks, saccades, body movements, EEG sleep spindles, etc.). A key step for data preservation and sharing is to annotate these experimental events using a standardized vocabulary rather than laboratory-specific or project-specific codes. In ESS, this is achieved by using new version 2.0 of the Hierarchical Event Descriptor (HED) tagging system we have developed (Bigdely-Shamlo et al., 2013b) to describe complex real-world events. The HED standard is freely available at *www.hedtags.org* along with graphical user interface tools that facilitate HED tagging of existing data (Rognon et al., 2013).

**FIGURE 2:**
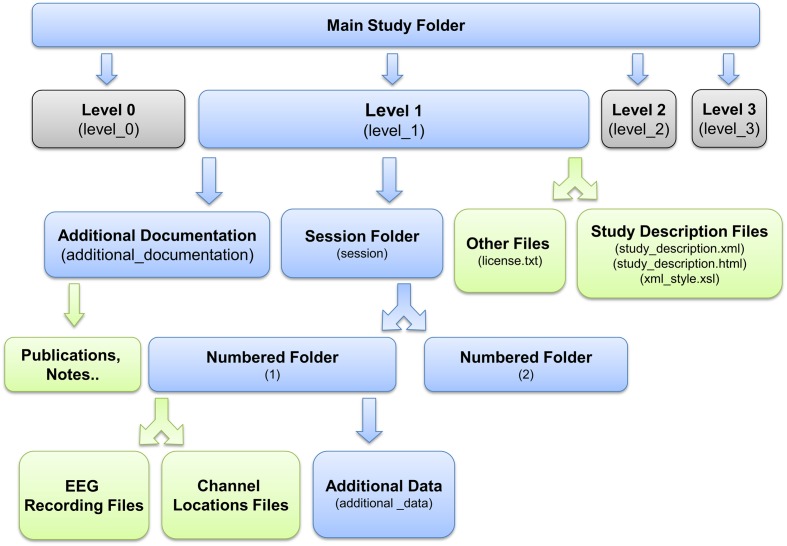
**EEG Study Schema *Standardized Data Level 1* folder and file structure (blue: folder, green: files)**.

After identifying events and mapping them to the EEG timeline, researchers must place study data into an ESS container, a standardized study metadata file together with a standardized arrangement and naming scheme for files and file folders (**Figure 2**). The ESS XML file holding study metadata includes meta-information including descriptions of the study paradigm and tasks. The file adheres to a predefined schema and encodes associations between sessions, subjects, groups, tasks, events, and data recordings. As part of building this file, researchers also specify the channel locations for the EEG data and also describe any other recording channels.

Once in this *Standardized Data Level 1* format, the data are fully containerized, allowing users to more completely automate subsequent processing steps by applying operations uniformly *across* containers without code revision. We have developed tools^12^ running on MATLAB (The Mathworks, Natick MA) to facilitate and validate the above process. We provide more details about the containerization process below.

**FIGURE 3:**
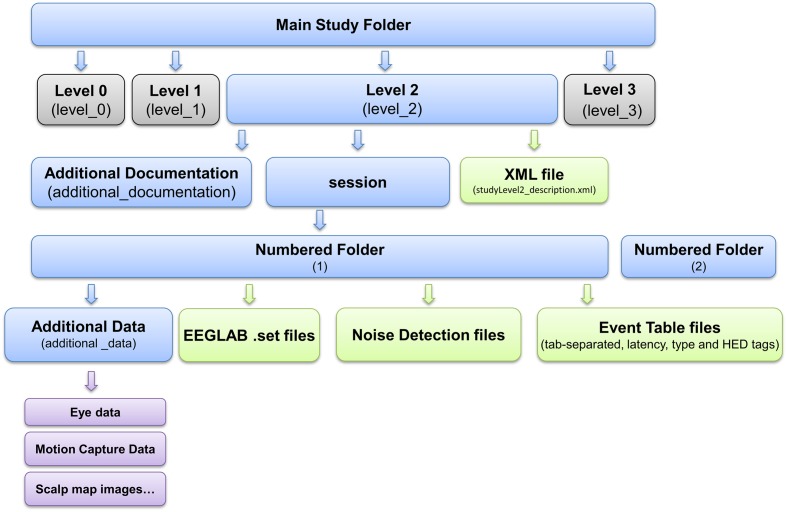
**EEG Study Schema *Standardized Data Level 2* folder and file structure (blue: folder, green: files, magenta: additional data)**.

*Standardized Data Level 1* provides a containerized interface to well-documented raw data. However, EEG data usually requires additional processing such as channel re-referencing before it is usable by most processing pipelines. We have therefore also developed a containerized specification and tools to automatically process and move data from raw *Standardized Data Level 1* to preprocessed *Standardized Data Level 2*. These tools apply the fully automated PREP software pipeline (Bigdely-Shamlo et al., 2015) to detect noisy EEG channels, to interpolate them using data from neighboring channels, and to transform the channel EEG data using a robust average reference. **Figure 3** shows the structure of a containerized *Standardized Data Level 2* study.

Note that placement of the study into *Data Level 2* is optional since data containerized in *Data Level 1* is already prepared and documented for sharing. The ESS toolkit also provides the ability to package additional processing steps using further *Level-derived* containers. This facility allows users to represent their own analysis pipelines as a series of containerized processing steps. The XML documents describing level-derived containers have a similar structure (e.g., nodes and schema) to the *Data Level 2* study description XML document and include the XML content of the parent container.

In particular, we envision possible *Standardized Data Level 3* specifications for *brain source-resolved* EEG data (Pascual-Marqui et al., 1994; Bell and Sejnowski, 1995; Makeig et al., 1996, 2004). Transformation of EEG data into a common brain source-space representation (cortical surface, volume, regions of interest, distributed source models or their equivalent dipole locations) can allow researchers to compare EEG dynamics occurring in different studies. Source-resolved data analysis can distinguish contributions from non-brain (artifact) processes such as line noise, eye blinks, muscle activities, etc., and effective brain source processes. It can also eliminate incompatibilities between studies and sessions arising from differences in the numbers and placement of the recording electrodes. Ultimately, it can facilitate interpretation of EEG brain source features by direct reference to functional brain atlases derived from fMRI and other functional imaging data. At least one method has been proposed for identifying equivalent cortical brain source processes across individuals taking into account major individual differences in cortical geometries (Tsai et al., 2014).

### Standard Level 1 Overview and Tools

We now give an overview of the ESS *Data Level 1* container structure and then describe how a researcher might map an existing study into such a container using supporting tools that make this process easier.

#### Level 1 Containers

**FIGURE 4:**
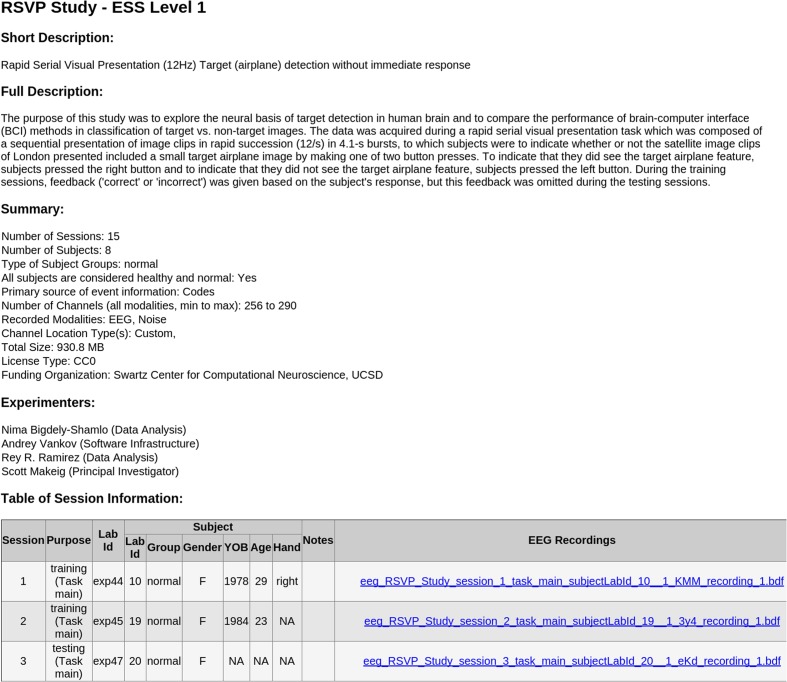
**Sample automated HTML report generated in the Chrome web browser by the included XSLT styling sheet from the *study_description.xml* manifest file of an ESS Standardized Level 1 container**.

A *Standardized Data Level 1* (*Data Level 1*) container has a specific folder structure (shown in **Figure 2**). The file *study_description.xml*, which is the “header” or “manifest” for the ESS container, contains the study metadata. The container itself has the following structure:

• [*Top-level documentation*]The documentation includes a README text file, the XML *study_description* file (the manifest), and a corresponding XSLT style sheet. Opening the study_descriptio.xml file in a web browser displays a formatted, easy-to-read, report generated by the XSLT file (see **Figure 4**). Due to the more stringent security rules in Google Chrome browser, this feature is not available for local files viewed in this browser.
• [*Additional information directory*] This directory contains publications and documents relevant to the study, including additional documentation about data and/or the task as well as unstructured experimental metadata (e.g., survey results, logs, etc.).
• [*Session directory*] The session directory contains a numbered subdirectory for each session. These subfolders hold one or more data recording files in their native raw format (e.g.,.*BDF*,.*EDF*, etc.) or in EEGLAB dataset (*.SET*) format (Delorme and Makeig, 2004). The subfolder also contains a channel location file for each subject (in its native raw format, also specified) unless the dataset indicates the recording used a standard montage (e.g., the International 10–20 System) without further electrode position information. The subfolder includes a tab-separated event-instance text file (event file) for each data recording. Each event file contains a list of all of the events in the associated recording, along with the event latencies (in seconds from the beginning of the recording) and their HED (version 2) annotation tags. This file allows accelerated, multi-platform access to event information without the need for reading large binary raw data files.

Raw data files, channel locations, and event files follow a naming convention that encapsulates session number, task label, and original file name. This naming convention makes it easier to distinguish files without referring to details in the XML manifest file.

#### Containerizing an Existing Study

This section presents a more detailed description of how an existing study might map to an ESS *Data Level 1* container. To prevent misunderstanding, we first define the terms *study*, *task*, *session, data recording*, and *recording parameter set*.

A ***study*** is a set of data collection efforts (recording sessions) aimed to answer one or a few, typically related scientific questions. The study data generally share the same or at most a few experimental paradigms.

A study ***session*** refers to all the data collected using a single EEG channel scalp montage from one subject (or from multiple subjects simultaneously, each using a single EEG montage). Typically, a researcher creates a session by securely mounting a cap containing EEG sensors to the scalp of a participant and recording data during some time interval (with or without breaks in recording).

A ***task*** within a study session is data recorded during a portion of a study session in which the subject or subjects are performing one or more (typically) well predefined sets of activities with specific goals. A study session may comprise data recorded during multiple experimental tasks and task conditions. For example, in a typical EEG study session, experimenters might ask participants to rest for 5 min with eyes closed (a.k.a. a baseline eyes-closed task), then to perform a computer-delivered visual target detection and response task, and finally to perform a specific ‘mind-wandering’ task. During each task, participants (typically) respond to environmental events with a well-defined set of rules and objectives. During “real-world” imaging sessions, however, tasks may not be explicitly defined, and an experiment session may consist of a single recording of EEG brain activity, behavior, and environmental events without clear task boundaries.

A ***data recording*** is a single data file containing continuous, synchronously recorded time-series data from one or more subjects. The recording file may contain non-EEG data channels such as galvanic skin response (GSR), ECG, body motion capture, and/or eye tracking data. Each channel often has a fixed recording rate. Some channels, such as channels containing events may have irregular sampling, e.g., only contain samples indicating subject button presses. Different sets of channels may have different recording rates. The channels, their modalities, and their sampling rates are identified by a single *recording parameter set*.

A ***recording parameter set*** characterizes the recording channels for one or more data recordings. The recording parameter set is organized into modalities, each consisting of a group of consecutively numbered recording channels that constitute a record from a particular modality recorded at a specified sampling rate. The specification includes channel names (e.g., standardized electrode locations, if available), measured channel locations (when available), and the system used to specify channel locations (when applicable).

To containerize a data collection, a researcher specifies how the collection maps to the above concepts by creating a *manifest*. This process usually involves editing an XML template and filling in the specifications for the *recording parameter sets* and the *data recordings*. The researcher then iteratively validates and corrects the manifest until it is error-free using ESS tools.

Alternatively, ESS tools enable creation of the manifest file using custom MATLAB scripts, though the manifest unavoidably requires some manual processing during its creation, The data manifest provides ESS with a description of the structure of the data, allowing ESS to (mostly) automate the downstream processing required after manifest creation. For example, after creating a proper manifest, researchers can use ESS tools to distribute the data into an appropriate folder/filename structure within the container.

We now present an example of how a researcher might create a manifest to map a data collection representing a single study into a container. The ESS standard accommodates more complex experimental designs, but the simplest use case illustrates the key ideas.

#### Example

Each of 24 participants performs an auditory oddball experiment replicating an earlier experiment (here, Squires et al., 1975) for approximately 15 min. The researchers use a Biosemi system to record 64 EEG channels and four external EOG channels at a data sampling rate of 1024 Hz.

The containerized version of this data set consists of 24 sessions, each of which contains a single data recording. Since all of the sessions use the same recording parameter set, the researcher defines a single *recordingParameterSet* element (*rset_1*) and 24 data *dataRecording* elements. The recording parameter set contains a single EEG modality that consists of 64 channels of scalp EEG and four non-scalp channels (with non-template/filled-in items highlighted in yellow):

**Figure:**
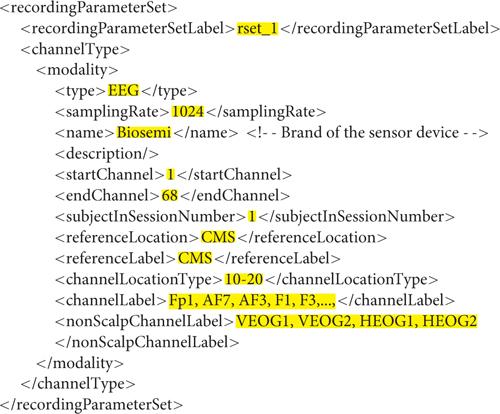


Each session has a number, a task label, optional subject metadata (such as gender, age, hand, height, weight, and medication), optional notes, and a list of data recordings. A data recording specification (with non-template/filled-in items highlighted in yellow) is shown below. A *dataRecording* element associates a particular EEG recording (*originalFileNameAndPath*) with a recording parameter set (*rset_1*).

**Figure:**
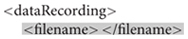


**Figure:**
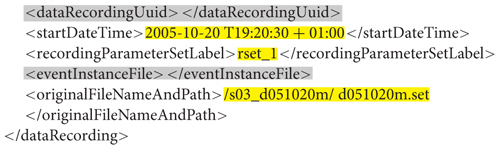


Researchers leave the grayed-out elements blank at this point for ESS to generate later. The ESS user’s guide^13^ gives a more detailed description of the manifest structure.

#### ESS Study Creation Tools

After creating a preliminary version of the XML manifest, researchers can take advantage of the ESS Toolbox^14^ for MATLAB to create ESS containers and the associated study files. The first step is to use the preliminary XML file to create a *level1Study* object in MATLAB:

**Figure:**
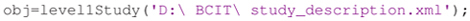


Next, to make sure the manifest contains all the necessary information and is self-consistent the researcher validates the manifest by calling the ESS validation function:

**Figure:**
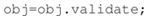


The validation function runs more than 30 checks and fixes minor issues such as missing UUIDs for data recordings. Failure to validate initiates an iterative process of validity checking and manifest correction that continues until ESS successfully validates the manifest.

At this stage, although the fields in the ESS *Data Level 1* MATLAB object (*obj*) have appropriate values, the files do not yet follow the ESS naming convention and the event instance files do not yet exist.

The final step for containerization is to create the folder structure with the manifest. The *createEssContainerFolder* method copies the data files from the original directory to a new container directory, renaming data recording files to adhere to ESS naming conventions and arranging them into separate session folders. It also generates event instance files from event instances in the data recording and HED tags associated with these events. For example, the following call copies the data from the original folder *D:∖BCIT* into the new container folder *D:∖BCIT_Level_1*:

**Figure:**
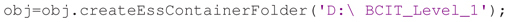


After this step, the data will be in a *Data Level 1* container described by a *study_description.xml* file with the file folder structure illustrated in **Figure 2**. All subsequent study levels and further study data processing (or cross-study meta-analyses) can be fully automated, since detailed study metadata is encapsulated at *Data Level 1* in a standardized manner.

#### Events in ESS *Data Level 1*

Events and event types play a central role in EEG processing, and the lack of consistent annotation of event markers in EEG data is a significant barrier for EEG data sharing. Traditionally, researchers mark events of a small number of types, labeling them using laboratory-dependent or study-dependent terminology. Often, researchers simply assign each type of event a numerical “code” during experimental setup. When an event of a specified type occurs, the experimental control software records the appropriate event code in a special “event channel” data stream time-locked to the recorded EEG data. Meaningful descriptions of these event numbers (or codes) are typically not available in the raw recording files. How researchers handle these raw “event codes” depends on the data analysis software they use. Using EEGLAB, for example, researchers typically map the event numbers (or codes), event latencies, and if available other event parameters into the *EEG.event* structure before further processing.

An alternative type of system, first introduced in (Bigdely-Shamlo et al., 2013b) is based on EEG experiment records produced by Lab Streaming Layer (LSL^15^) in Extensible Data Format (XDF^16^). This system records a data stream of ascii strings that characterize the experiment events using the Hierarchical Event Descriptor (HED) system. The experimental control software composes event strings and delivers them, at times of occurrence, into the data stream, time-locked to the EEG and any other concurrently recorded time series data streams. HED event strings can encode significantly more information than simple event type numbers and thereby can provide a much richer description of experimental events. Such a system is particularly useful in more complex experiments in which the potential events and their combinations cannot be represented using a small number of pre-defined event codes. Moreover, such an event description system is essential to performing data meta-analysis across studies collected seprately. The number of potential combinations of terms from a predefined annotation vocabulary can be extremely large; in some experiments each composed event string may even be unique. However, the HED structure makes the events easy to select or sort on any attribute specified in the HED strings, without needing to refer to some external table, investigator notebook, or publication.

Regardless of how events are acquired, stored, and documented, researchers using ESS must map their descriptions into a standardized vocabulary. This standardization allows others to understand the meaning of the events. It also allows a scientist to analyze their data and its events within a larger context of other experiments and their events, or to perform meta-analysis across independently collected studies.

EEG Study Schema uses the HED 2.0 (Hierarchical Event Descriptor) vocabulary for annotating events. The detailed specification of this annotation scheme and supporting tools are available at www.hedtags.org. Because HED is hierarchical, researchers can annotate events using as much or as little detail as they choose. Each event must have a label (20 characters or less) and a category. The label is experiment-specific. If they choose, researchers may use their standard laboratory event codes for the labels.

For example, a simple annotation for the auditory oddball experiment (Squires et al., 1975) used in the example above may identify only two types of events, both with an event category of ‘experimental stimulus’ (i.e., stimulus presentation):

**Figure:**
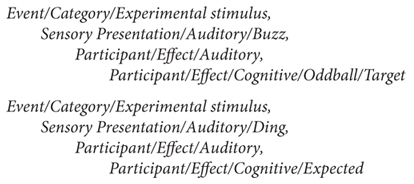


These tags indicate that an auditory stimulus (either *Sensory Presentation/Auditory/Buzz* or *Sensory Presentation/Auditory/Ding*) was presented and (may be presumed to have been) heard by the subject (*Participant/Effect/Auditory*). The ding was expected (*Participant/Effect/Cognitive/Expected*), while the buzz was a less expected ‘Oddball’ that served as a subject detection target (requiring some subject response) (*Participant/Effect/Cognitive/Oddball/Target*). If the subject, for example, pressed one of two buttons to indicate which stimulus type was perceived, the experimenter would add an additional event code and corresponding tags to describe the type (and, automatically, the latency) of the participant response event.

The HED tagging vocabulary supports event annotations at an arbitrary level of detail. Tools to facilitate these annotations and to integrate HED tagging into containerized ESS studies are available on the HED website^17^. Once a researcher creates a mapping of experimental event types or codes to HED tags via HED annotation tools (else manually using a spreadsheet or other method), ESS embeds this mapping as metadata in the container XML manifest in the form of a table that specifies the HED tags associated with each event code or label for each task.

### Standardization Beyond Basic Containerization

Putting data into ESS *Standard Data Level 1* makes the data sharable. However, most researchers perform many processing or pre-processing steps on the EEG data before final analysis. To facilitate these efforts and to make the results shareable, we have developed additional containerization functions that allow researchers to apply their own data processing pipelines to a containerized data collection, thereby producing a new “processed data container.” The resulting data container also contains the data processing command used to produce it in a standard API form, allowing users to apply the same pipeline to different containerized collections without code modification.

Because of the need for most researchers to reference and identify artifact-dominated (bad) channels, we have developed *Data Level 2* schema and related ESS tools to provide a containerized, well-documented version of the data after processing by a fully automated and standardized pre-processing pipeline. The pipeline also provides an extensive summary report of data quality, of use for properly assessing results obtained from studies comprised of data sets of differing quality. Our *Data Level 2 schema* uses the PREP pipeline (Bigdely-Shamlo et al., 2015), which detects bad channels dominated by experimental artifacts and performs robust re-referencing. PREP can be run as part of ESS, as a standalone procedure, or from EEGLAB. *Data Level 2* processing is optional, and the data is distributable as a *Data Level 1* container. Given a container at any level, researchers can also apply their own processing and produce a custom derived container, as described later in this document.

**Figure 3** shows the layout of the data container resulting from *Data Level 2*. For more details, see eegstudy.org/schema/level2. Note that creation of a *Data Level 2* container from a *Data Level 1* containerized collection is entirely automated and can be performed with no user intervention:

**Figure:**
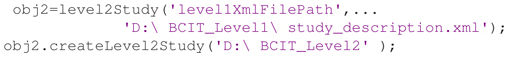


where *D:∖BCIT_Level1∖study_description.xml* is the *Data Level 1* manifest.

*Level-derived* containers allow execution of customized processing scripts on all the recordings in an ESS container, containerizing the results for downstream automated processing. Researchers can create *Level-derived* containers from other *Level-derived* containers, resulting in a chain of containerized results that encapsulates a completely documented workflow. A *Level-derived* container has a file and folder structure similar to a *Data Level 2* container and may include auxiliary reports such as data quality estimates and results of data processing. For more details, see *eegstudy.org/schema/levelDerived.*

Using a containerized approach requires three steps: (1) creating a *levelDerivedStudy* object; (2) defining a processing function in the appropriate format; and (3) creating the container. Following is sample code to create a *Level-derived* container from a *Data Level 2* container that implements processing the data using a *highPass* filter function with a *detrendCutoff* parameter value of 0.5:

**Figure:**
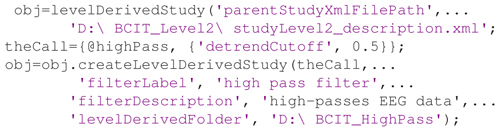


Computationally, using EEGLAB processing functions, the above pipeline is equivalent to iterating the following MATLAB code over all data recordings in the container:

**Figure:**
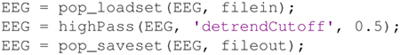


However, the *Level-derived* code creates a fully containerized data collection with a manifest so that researchers can chain processing steps together in the containerized format. Researchers do not have to be concerned with the names or directory structure of the recordings within the container; ESS applies the specified function to every recording in the container. The current version of ESS tools requires the EEG datasets to be in EEGLAB *EEG* structure format to compute derived measures. Note: Data Level 1 containerization does not require EEGLAB structures. However, when a user applies the *Data Level 2* fully automated pipeline, ESS saves the resulting datasets as EEGLAB *EEG* structures.

To specify a derived measure, researchers define a function that receives an *EEG* structure as input, along with parameters in name-value pairs, and outputs another *EEG* structure. ESS containerizes the resulting datasets as EEGLAB.*set* files. Future options may incorporate other input and output formats for the EEG *Level-derived* containers.

## Results

We have added pointers to over 460 data session recordings in an online catalog of containerized studies that are now available (under various terms) in ESS format^18^ These include three driving-related studies collected under the CaN-CTA Real-World Neuroimaging Project (McDowell et al., 2013), 80 sessions of a driving lane-keeping study (Lin et al., 2005) and 15 sessions using a Rapid Serial Visual Presentation (RSVP) paradigm (Bigdely-Shamlo et al., 2008) into *Standardized Data Levels 1&2*. We have also placed data from 18 studies (381 subjects) into *ESS version 1.0* containers and are in the process of converting these containers to ESS version 2.0. Seven of these studies are publicly available at headit.org. Appendix B (Table B1) summarizes our containerization efforts to date (853 GB of raw data total). As a validation of the approach, we have successfully performed a number of secondary analyses using the ESS infrastructure across these collections without issues.

We are continuing to containerize a large number of other studies and to develop additional tools that operate on containerize collections^19^. We intend for the Study Catalog site^18^ to be a central catalog of pointers to an ever-growing number of containerized EEG studies potentially available (under various terms and conditions as determined by their authors) for analysis and meta-analysis projects.

## Discussion

Automated EEG analysis requires access to specific information, such as the type of referencing, recording channels that are not on the scalp (e.g., to be excluded from the average reference), exact channel locations or the standard channel location montage used, etc. To our knowledge, ESS is the only schema that provides this information. Another unique feature is that ESS provides containers for EEG data sets in different stages of analysis, along with a method for documenting data provenance using ESS filters. Since ESS is a schema for study metadata encapsulation, among technologies listed in **Table 1** it is only comparable to ISA-TAB and odML. However, ISA-TAB and odML are general in nature and require EEG domain-specific schemas to be built on them to provide the functionality of ESS.

Containerization is an important concept for efficient handling and processing of EEG. To understand its advantages, consider the process of making use of data stored in a non-containerized format, for example Physionet, which does have some EEG studies. The maintainers of Physionet have exerted considerable effort to make these files available in standardized form. However, to use them, investigators must download a large number of individual files and write code specific to each study. In contrast, researchers could download an entire ESS container from a single zip file and then process all container datasets with a few lines of code in MATLAB or other data processing environments. Furthermore, having developed code relative to the ESS interface, a researcher may apply it without modification across containers, e.g., across any or all studies saved and available in ESS format.

EEG Study Schema containerization addresses a specific set of data sharing issues by allowing researchers to access data and metadata using a standardized application program interface (API). The containerized format promotes easy data download and exchange using standard file transfer protocols. ESS does not contain any field for subject identifying and privacy-sensitive subject information such as name, social security number, address, email, phone number. Instead it uses lab-specific, anonymized subject codes. We assume that the ESS exchange mechanism will be used primarily for publicly released de-identified data; a more explicit privacy policy enforcement is not within the present scope of ESS.

EEG Study Schema does not provide any standardized format for clinical data; it currently provides a standard format for a subset of subject characteristics including gender, age, and handedness (clinical group status indications not included, though these can be added as needed). ESS also does not include any specific mechanism for maintaining data security or integrity, but rather assumes the standard mechanisms for maintaining file integrity provided by the hosting file systems are sufficient. ESS assumes that containers are uncompressed during analysis, but researchers are free to compress the containers for exchange or for hosting.

Finally, ESS is not a high-performance computation (HPC) mechanism but can facilitate parallel processing of EEG data through metadata encapsulation, e.g., ESS containers are good candidates for “embarrassingly parallel” workloads since in many cases data from each session can be processed independently (e.g., Map operation in the MapReduce programming model Dean and Ghemawat, 2008). ESS provides a mechanism for processing subsets of container datasets and then recombining the results into a single container (as in the Reduce operation in the MapReduce programming model).

One limitation of our current tools is the still unmet need for easy manual or semi-automated generation of Level 1 manifest file in XML format. Currently, users must type this XML file and perform iterative validation steps. Alternatively, they must write custom MATLAB scripts to import the metadata from semi-structured formats such as spreadsheets and make use of ESS tools to place these data in the XML file. An intelligent data import GUI (Wizard) could make this step more user-friendly.

Also, ESS encapsulates metadata in XML while JSON (Bray, 2014) is currently the more popular and faster (Nurseitov et al., 2009) data encapsulation format for web technologies. In addition, modern databases (e.g., MongoDB and PostgreSQL 9.2+) provide better support for querying JSON documents. We plan to provide extensive ESS input/export support for JSON in near future.

## Future Directions

The investment in the annotation of fMRI data (Laird et al., 2005; Poldrack et al., 2013) has paid huge dividends in allowing meta-analysis and new insights from existing data (e.g., Houdé et al., 2010; Xu et al., 2015; Poldrack and Yarkoni, 2016). We believe EEG meta-analysis can reach a similar level of productivity and impact, providing new insights into brain patterns and behavior across populations while also allowing researchers to assess the degree and type of individual subject variability in EEG brain dynamics occurring when different subjects perform the same and/or similar tasks.

To this end, we intend to extend the ESS/HED technology stack by creating additional user-friendly tools and algorithms that facilitate data discovery and large-scale inference, particularly using source-resolved data – for an example see (Bigdely-Shamlo et al., 2013a).

Creating exporters from ESS to ISA-Tab and linked-data using JSON Linked-Data, JSON-LD (Sporny et al., 2013), and the NIDM-Experiment ontology (Nichols and Maumet, 2016) would enable accessibility of EEG metadata to a wider set of tools for search and discovery.

Tighter integration between ESS and EEGLAB and/or other open source EEG analysis environments, as well as commercial EEG analysis tools such as BrainVision Analyzer (Brain Products GmbH) and Brain Electrical Source Analysis (BESA) could improve the interoperability between these environments.

Parallel execution of PREP and other pipelines on ESS container recordings would accelerate large-scale EEG processing.

Automated registration of ESS files in a central study catalog^20^ could enable EEG researchers to make their metadata public, while optionally keeping their data available only on terms they specify.

The study catalog would allow other researchers to find these studies and to contact data owners concerning data-sharing terms, thus forming a scientific listing service for EEG data.

Advancing EEG brain imaging research to make use of powerful ‘big data” methods is in some ways a “chicken and egg” problem: Why should funders fund and researchers format and contribute data and data descriptions unless proven analysis tools are also available to readily extract more information from the data? On the other hand, availability of data annotation and aggregation tools alone is not likely to attract active participation of research users. The basic software infrastructure described in this paper is a first step in making automated large-scale EEG data analysis a reality. Community contributions of data, development of meta-analysis tools, and convincing demonstrations (at some feasible size scale) are needed to make EEG data meta-analysis widely available. We believe that such a prospect should stimulate research community excitement sufficiently to gain and demonstrate broad support for better informed analysis and meta-analysis of EEG data and welcome discussions with others who have similar or related interests in achieving these goals. We believe ESS to be an important first step in this direction, and have accordingly made all of our tools freely available at eegstudy.org under MIT license.

## Author Contributions

NB-S devised ESS and HED technologies, wrote ESS Tools, wrote parts of the paper and made the final revision. KR wrote large portions of the paper, worked on ESS and HED tools with NB-S and made several revisions. SM contributed to the inception of ESS and HED. He also made several significant revisions and additions to the paper.

## Conflict of Interest Statement

The authors declare that the research was conducted in the absence of any commercial or financial relationships that could be construed as a potential conflict of interest.

## Footnotes

1 linuxcontainers.org

2 docker.com

3 bids.neuroimaging.io

4 incf.org/resources

5 http://www.isa-tools.org/

6 figshare.com

7 eegdatabase.kiv.zcu.cz

8 headit.org

9 bigeeg.org

10 headit.org

11 www.eegstudy.org

12 github.com/BigEEGConsortium/ESS

13 eegstudy.org/schema/level1

14 github.com/BigEEGConsortium/ESS

15 github.com/sccn/labstreaminglayer

16 github.com/sccn/xdf

17 www.hedtags.org

18 studycatalog.org

19 www.eegstudy.org

20 studycatalog.org
