# Supplementary material for: Preparing Laboratory and Real-World EEG Data for Large-Scale Analysis: A Containerized Approach
Source: Front Neuroinform. 2016 Mar 8;10:7. doi: 10.3389/fninf.2016.00007 (PMC4782059; doi:10.3389/fninf.2016.00007)
Supplement: Supplementary file 1 [file Appendix.DOCX]

**APPENDIX A**

**Table A.1, List of online resources in use in the BigEEG project to make standards and software available.**

| **URL** | **Description** |
| --- | --- |
| *BigEEG.org* | BigEEG Consortium website |
| *HedTags.org* | Hierarchical Event Description (HED) schema and tools, for event tagging |
| *EegStudy.org* | EEG Study Schema (ESS) schema and tools, for study  containerization |
| *StudyCatalog.org* | Central catalogue of pointers to an ever-growing number of containerized EEG studies available under various terms and conditions specified by their authors |
| *vislab.github.io/…*  *EEG-Clean-Tools* | PREP pipeline for noisy channel detection and robust referencing |
| *github.com/sccn …*  */labstreaminglayer* | Lab Streaming Layer (LSL) , for transmission of multi-modal data |
| *github.com/sccn/…*  *xdf* | Extensible Data Format (XDF) for storing multi-modal data |

**APPENDIX B**

**Table B.1, List of studies in ESS format.** The column titles: ESS V. = ESS version; L1 indicates whether the data is available in Standardized Level 1; L2 indicates whether the data is available in Standardized Level 2; S = number of sessions. For an up-to-date study list with links, please visit *studycatalog.org.*

| **Study** | **ESS V.** | **L1** | **L2** | **S** | **Raw Size**  **GB** |
| --- | --- | --- | --- | --- | --- |
| **RSVP Target Detection**  (Publicly available at studycatalog.org)  Presents bursts of 12/s satellite image clips, some with an embedded target airplane image, without immediate button press | 2 | Yes | Yes | 15 | 14 |
| **NCTU Lane-keeping**  (Sharable with Collaborators)  Drowsiness study with lane perturbation and response in a simulated car driving with and without VR-controlled motion. | 2 | Yes | Yes | 80 | 28 |
| **ARL Traffic Complexity**  (Sharable with Collaborators)  Evaluates whether increasing visual complexity of the driving environment affect's driver alertness and its regression-based estimation | 2 | Yes | Yes | 28 | 16 |
| **ARL X6 Speed Control**  (Sharable with Collaborators)  Evaluates whether the degree of interaction with the simulated vehicle, i.e., manual versus automatic control of vehicle speed, would affect driver alertness and its estimation | 2 | Yes | Yes | 24 | 26 |
| **ARL XB Baseline Driving**  (Sharable with Collaborators)  15-minute baseline driving sessions associated with all of the research areas specified within the Army Research Laboratory | 2 | Yes | Yes | 108 | 267 |
| **ARL X1 Baseline RSVP**  (Sharable with Collaborators)  Investigates whether performance of a non-driving task (RSVP) could be predicted using spectral data from electrodes overlying central-parietal regions | 2 | Yes | Yes | 23 | 82 |
| **ARL X2 RSVP Expertise**  (Sharable with Collaborators)  Investigates whether performance of a non-driving task (Expertise RSVP) could be predicted using spectral data | 2 | Yes | Yes | 43 | 151 |
| **ARL X3 Baseline Guard Duty**  (Sharable with Collaborators)  Investigates whether performance of a non-driving task (Guard Duty) could be predicted using spectral data from electrodes overlying central-parietal regions | 2 | Yes | Yes | 18 | 48 |
| **ARL X4 Advanced Guard Duty**  (Sharable with Collaborators)  Investigates whether performance of a more complex non-driving task (Advanced Guard Duty) could be | 2 | Yes | Yes | 20 | 50 |
| **ARL XC Calibration Driving**  (Sharable with Collaborators)  15-minute calibration driving sessions associated with all of the research areas specified within the Army Research Laboratory | 2 | Yes | Yes | 196 | 112 |
| **Auditory-Visual Attention Shift**  (Publicly available at Headit.org)  Young and older adults perform a visual-auditory cued attention shift paradigm. | 1 | Yes | No | 49 | 1 |
| **Auditory Two-Choice**  (Publicly available at Headit.org)  Equally probable longer and shorter tones were so categorized by subjects | 1 | Yes | No | 12 | 8 |
| **Reward Two-Back CPT**  (Publicly available at Headit.org)  Visual two-back Continuous Performance Test with auditory feedback | 1 | Yes | No | 25 | 11 |
| **Modified Sternberg Working Memory Task**  (Publicly available at Headit.org)  Visual letter memory task (recall black letters; ignore green) | 1 | Yes | No | 23 | 5 |
| **Imagined Emotion**  (Publicly available at Headit.org)  Subjects listened to voice recordings that suggest an emotional feeling and ask subjects to imagine an emotional scenario | 1 | Yes | No | 32 | 34 |
| **Five-Box Task**  (Publicly available at Headit.org)  A visual spatial selective attention oddball task. | 1 | Yes | No | 60 | 2 |
